# Genomic assays for Epstein–Barr virus-positive gastric adenocarcinoma

**DOI:** 10.1038/emm.2014.93

**Published:** 2015-01-23

**Authors:** Margaret L Gulley

**Affiliations:** 1 Department of Pathology and Laboratory Medicine and Lineberger Comprehensive Cancer Center, University of North Carolina at Chapel Hill, Chapel Hill, NC, USA

## Abstract

A small set of gastric adenocarcinomas (9%) harbor Epstein–Barr virus (EBV) DNA within malignant cells, and the virus is not an innocent bystander but rather is intimately linked to pathogenesis and tumor maintenance. Evidence comes from unique genomic features of host DNA, mRNA, microRNA and CpG methylation profiles as revealed by recent comprehensive genomic analysis by The Cancer Genome Atlas Network. Their data show that gastric cancer is not one disease but rather comprises four major classes: EBV-positive, microsatellite instability (MSI), genomically stable and chromosome instability. The EBV-positive class has even more marked CpG methylation than does the MSI class, and viral cancers have a unique pattern of methylation linked to the downregulation of *CDKN2A (p16)* but not *MLH1*. EBV-positive cancers often have mutated *PIK3CA* and *ARID1A* and an amplified 9p24.1 locus linked to overexpression of *JAK2*, *CD274* (*PD-L1*) and *PDCD1LG2* (*PD-L2*). Multiple noncoding viral RNAs are highly expressed. Patients who fail standard therapy may qualify for enrollment in clinical trials targeting cancer-related human gene pathways or promoting destruction of infected cells through lytic induction of EBV genes. Genomic tests such as the GastroGenus Gastric Cancer Classifier are available to identify actionable variants in formalin-fixed cancer tissue of affected patients.

## Introduction

Epstein–Barr virus (EBV) is present within the malignant cells in ~9% of all gastric adenocarcinomas worldwide.^1^ Another pathogen, *Helicobacter pylori* (*Hp*), commonly present in the stomach, confers a fivefold increased cancer risk.^2^ Gastric cancer is the leading cause of infection-related cancer mortality and is projected to soon rise to rank eighth in all-cause mortality.^3, 4^ By the time gastric cancer is diagnosed, a billion or more malignant cells are typically present harboring dozens to thousands of somatic gene variants. Genomic tests show promise to identify driver mutations such as *ERBB2* amplification for which targeted therapy may be effective.^5^ Understanding the functional impact of genomic variation promotes selection of drugs that target relevant pathways and thus could overcome unwanted effects that promote growth, thwart apoptosis, elude the immune system or impair DNA repair mechanisms that foster more mutagenesis.^6, 7, 8, 9^

Enrollment in clinical trials of targeted therapy increasingly relies on results of tests for pertinent genes and gene products. This article describes the current state of genomic assay development for gastric cancer and the opportunities to capitalize on EBV and its effectors as targets for therapy.

## Four major molecular classes of gastric adenocarcinoma

Data from The Cancer Genome Atlas (TCGA) Network suggest that gastric adenocarcinoma is not one disease but rather comprises four molecular classes, as discovered by comprehensive tests of 295 frozen gastric cancer tissues from untreated patients using whole exome sequencing, RNAseq, microRNA sequencing, SNP array, methylation array, reverse-phase protein array, microsatellite instability (MSI) testing and (in 107 tumors) whole genome sequencing.^10^ Matched germline DNA (generally from blood) and non-malignant gastric mucosa were also examined. The summary of key findings is provided in Table 1.

## MicroRNA and messenger RNA profiles in EBV-positive gastric adenocarcinoma

EBV was the first virus recognized to encode its own microRNAs. MicroRNA and mRNA profiles are achievable in formalin-fixed, paraffin-embedded tissue using massively parallel sequencing or array technology. The summary of RNAs distinguishing each of the four cancer classes from non-malignant gastric mucosa is provided in Table 2.^10, 11, 12, 13, 14, 15, 16, 17, 18^ Several downregulated mRNAs are shared among the four gastric cancer classes, implying these RNAs are pancancer markers in the stomach. Conversely, *CST1* is upregulated in all four cancer classes.

## EBV-associated DNA hypermethylation

A striking feature of EBV-positive gastric cancer is extreme CpG hypermethylation, including both promoter and non-promoter CpG islands of the human genome.^10, 19, 20^ Notably, the pattern of methylation is even more extensive than the classic CpG island methylator phenotype observed in the MSI class of gastric cancers, and is more extensive than was seen in any tumor type previously studied by the TCGA Network.^10, 21, 22^ Furthermore, EBV and MSI methylation patterns are distinct, with EBV-positive tumors displaying *CDKN2A* (*p16*) promoter hypermethylation but lacking *MLH1* hypermethylation.^10, 23, 24^

The genes silenced in virtually all EBV-positive cancers in concert with promoter hypermethylation are listed in Table 3.^10^ The *RCOR2* gene exhibited methylation-related silencing in 100% of EBV-positive and in 0% of EBV-negative gastric cancers. Work on cell lines suggests that downregulation of the *RCOR2* transcription factor promotes hypermethylation, whereas expression of *RCOR2* promotes reprogramming to stem cell pluripotency.^25, 26^

EBV infection leads to extensive methylation of both host and viral genomes, providing a mechanism for viral control of cellular functions promoting viral persistence and propagation.^20, 27, 28, 29, 30, 31, 32^ EBV BZLF1 was the first protein ever shown to preferentially bind methylated promoters to induce gene expression, thus overcoming transcriptional silencing to switch an infected cell from viral latency to active, lytic viral replication. Interestingly, *Hp* infection is also associated with hypermethylation.^33^ *In vitro* evidence points to demethylating drugs that can reverse the effect, but clinical trials of EBV-positive tumors (lymphoma and nasopharyngeal carcinoma) treated with 5-azacytidine plus phenylbutyrate had disappointing results.^34^

## EBV-directed therapy, and genomic tests to monitor efficacy

As viral DNA and selected viral gene products (see below) are present in every malignant cell of an EBV-positive tumor, a compelling cure for cancer would be to eliminate all infected cells. Strategies for virus-directed therapy are listed in Table 4.

**Figure 1:**
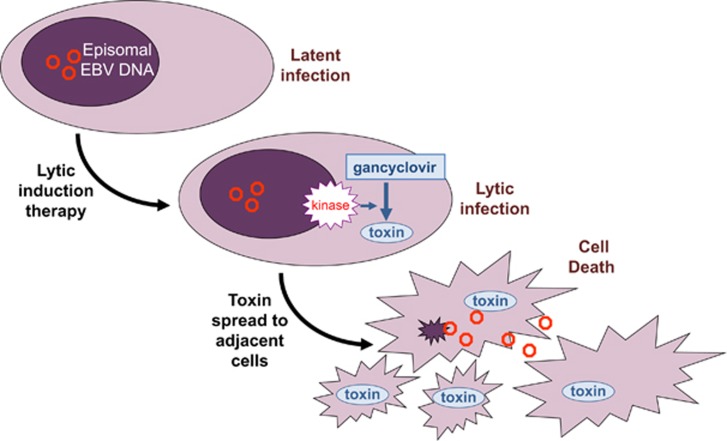
During latent infection, a very limited repertoire of viral gene is expressed. However, when an infected cell switches into the lytic phase of active viral replication, dozens of viral proteins are expressed that trigger immune recognition and destruction. Cell death may be enhanced by administering the anti-viral drug gancyclovir, a purine analog that is incorporated into DNA strands by DNA polymerase. Viral thymidine kinase (BXLF1) and serine/threonine protein kinase/phosphotransferase (BGLF4) phosphorylate gancyclovir, which then stalls DNA synthesis and triggers apoptosis. Intercellular transfer of phosphorylated gancyclovir can result in death of adjacent cells.

Lytic induction therapy is a rational means to promote destruction of infected cells, and the putative mechanisms of action were recently summarized by Kenney and Mertz.^35^ Radiation therapy and selected drugs induce lytic viral gene expression, enhancing immune recognition of foreign proteins.^35, 36, 37^ Radiation is effective in treating some EBV-infected cancers such as nasopharyngeal carcinoma and Hodgkin's lymphoma. Histone deacetylase inhibitors are among the most potent inducers of active viral replication.^38^ Short-chain fatty acids such as butyrate are also good inducers having reasonable safety profiles. Butyrate is produced naturally by certain bacteria comprising the oral and gastric flora.^39^ Nucleoside analog drugs such as gancyclovir may enhance cell death during lytic induction therapy^35^ (see Figure 1). Two clinical trials reported positive biologic effects and minimal toxicity.^40, 41^

A major aim of the lytic induction therapy is to provoke host cell expression of immunogenic foreign proteins that incite immune responses. Decades of experience treating EBV-driven posttransplant lymphoproliferation shows that cutting back on iatrogenic-immunosuppressive drugs restores the body's natural ability to control EBV infection, potentially reducing tumor burden as reflected by lower viral load in the plasma.^42^

In solid tumor patients, EBV-directed T cells infused in concert with immune modulators have some efficacy against infected cancers.^43^ Drugs that were ineffective as single agents are now being considered in combination, such as a histone deacetylase inhibitor (e.g. vorinostat, suberoylanilide hydroxamic acid or valproate) plus the proteasome inhibitor bortezomib.^35, 44, 45, 46, 47, 48, 49^ Triple drug therapy with gancyclovir plus gemcitabine and valproate (both of which induce lytic viral replication) showed anecdotal value in stabilizing three nasopharyngeal carcinoma patients.^41^ Clinical trials should report EBV status as well as pertinent genomic features to characterize exceptional responders and to shed light on mechanisms of action in relevant biochemical pathways.

Laboratory tests can measure the degree of lytic induction using quantitative PCR to quantify viral genomes and transcriptome profiles to measure lytic mRNAs. Parallel tests of *human* RNA evaluate the impact on pertinent cellular biochemical pathways. These tests may be applied to biopsy material, although periodic rebiopsy is impractical and risky. Plasma is emerging as a more practical specimen type in which to measure tumor markers (EBV viral load, somatic mutations and microRNA or methylation profiles) to assess near-term effects of intervention and long-term tumor burden.^50, 51, 52, 53^

## Host gene mutation

A salient feature of EBV-positive gastric cancer is *PIK3CA* mutation, which is found in 80% of such cancers compared with only 3–42% for cancers in the other three molecular classes. Interestingly, nearly half of EBV-positive nasopharyngeal carcinomas harbor *PIK3CA* mutation, implicating a common theme to viral carcinogenesis despite squamous *vs* glandular histologies. This is a classic example of genomic features linking two cancer types despite different histologies and anatomic sites.^54^

In EBV-positive gastric cancer, *PIK3CA* mutations are not restricted to hotspots (helical or kinase domains) but rather are spread across many gene segments.^10^ Clinical trials of various PIK3CA/AKT/mTOR inhibitors (e.g. everolimus, BEZ235) showed disappointing results as single agents in gastric cancer, and dual pathway inhibition is now being explored.^55, 56, 57, 58, 59, 60^ Unique cancer prevention strategies are also being explored for *PIK3CA*-mutated cancers.

Human genes commonly mutated in gastric cancer are listed in Table 5.^10^ The vast majority of cancers have mutation or gene amplification in a targetable signaling pathway (receptor tyrosine kinases such as *MET* or *EGFR*, *JAK/STAT*, GTPase *(RHO/RAS/RAF)*, *PIK3CA/MTOR/PTEN*, *CTNNB1*). RNAseq revealed *MET* exon skipping (of exons 2, 18/19 or 19) associated with overexpression of the encoded receptor. *MET* gene amplification was found in about a third of EBV-positive cancers.

EBV-positive cancers typically lack *TP53* mutation, although *TP53* was nearly always mutated in ‘chromosome instability' cancers,^10^ which might be detectable by immunohistochemical evidence of TP53 protein accumulation.^61^ Indeed, TP53 immunostains already serve as an adjunct to histopathology in predicting progression of Barrett's lesions to cancer.^62^ Shimizu *et al.*^63^ recently reported *TP53* or *ARID1A* mutation at low allele frequency in non-malignant gastric mucosa of patients with *Hp* infection, and *Hp*-related mutagenesis is purportedly related to activation of cytidine deaminase leading to characteristic C>T transversions of GpCpX motifs.

## EBV-related immune system dysfunction identified in TCGA studies

Autocrine or paracrine growth factors seem to promote growth of tumor cells. The TCGA Network reported that, compared with EBV-negative cancers, EBV-positive cancers have evidence of hyperactive adaptive and innate immunity, with evidence of T-cell activation via the cytokines interleukin-2 (IL-2), IL-12, IL-23 and IL-27.^10^ Some T-cell activation evidence undoubtedly emanates from tumor-infiltrating lymphocytes that tend to be abundant in infected cancer tissues, yet are unable to control growth of infected tumor cells.^64^ Other features of EBV-positive cancers are (1) diminished glucocorticoid signaling, suggesting an opportunity to test dexamethasone and other immune modulators, (2) defective cell adhesion and (3) strong caspase activity that might be exploited to tip the balance towards death of infected cells.

When compared with non-malignant gastric mucosa, EBV-positive cancers exhibit many of the same biochemical features as uninfected cancers, including strong DNA damage response pathways. A major difference is that interferon-γ and IFN-γ-induced interferon regulatory factor-1 in the IL-2-STAT4 pathway are overexpressed in EBV-positive cancers versus uninfected cancers.^65^

## EBV-related receptor kinase signaling

Compared with non-malignant mucosa, TCGA investigators reported that the two most marked features of EBV-positive cancers are diminished hypoxia-inducible factor 1α-related activity and diminished ERBB receptor signaling. These findings raise the question of whether angiogenesis inhibitors or ERBB family inhibitors might have differential efficacy in infected *vs* uninfected cancers. Pending further studies, it seems reasonable to continue to follow customary clinical recommendations^66^ regarding use of drugs such as ramucirumab targeting vascular endothelial growth factor receptor-2 involved in angiogenesis^67, 68, 69^ or trastuzumab in the setting of ERBB2 (HER2) overexpression/amplification.

EBV-positive cancers have evidence of activated BMP (bone morphogenetic protein) signaling, implicating that the BMP/SMAD pathway as a potential therapeutic target. In addition, potentially druggable *JAK2* or *MET* gene amplifications are relatively frequent among EBV-positive cancers.^7^ Intriguing studies show EBV-positive gastric cancers preferentially overexpress *CD274* and *PDCD1LG2* (*PD-L1* and *PD-L2*) as revealed by RNAseq and by protein localization to malignant cells by immunohistochemistry.^10, 70^ Prior studies of lymphoma likewise revealed EBV-associated upregulation of *CD274* on the cell surface, which is IFN-γ-mediated, and thus inhibits killing of infected cells by cytotoxic T cells expressing PD-1 ligand.^71^ Gain of four or more copies of the *CD274* and *PDC1LG2* genes, or mutation in the 3′-untranslated region of *CD274*, are alternative mechanisms of overexpression.^72^ PDCD1LG2 thwarts T-helper type 2 T-cell function. Further work is needed to explore virus-related immune evasion mechanisms, particularly now that PD/PD-L inhibitor drugs are available.

Regardless of EBV status, most gastric cancers have at least one aberrancy in a druggable pathway such as receptor tyrosine kinase signaling.^10, 73, 74, 75, 76, 77, 78, 79^ Newly identified in ‘genomically stable' gastric cancers is activated RHOA singaling via mutation of RHOA GTPase, or fusion events in RHOA inhibitors (*ARHGAPs*), broadening the opportunity to test inhibitors of the RHOA effector ROCK that are well studied in vascular biology. ROCK functions as a serine/threonine kinase impacting CDH1-mediated cell adhesion, tumor microenvironment and actin structural biology.^10, 80, 81^ CDH1, RHOA and ARHGAP defects are common and are mutually exclusive. *Hp* infection reportedly activates members of the RHO family of GTPases.

The *CD44* gene is preferentially amplified in EBV-positive cancers, and other commonly amplified or deleted genes are listed in Table 6. Previously identified were a CD44 protein variant or a CD44-SLC1A2 or SLC34A2-ROS1 fusion in cancer tissues.^82, 83^ Fusion genes are technically difficult to find by next-generation sequencing, and expensive to identify by fluorescence *in situ* hybridization. However, once identified as a somatic variant, a fusion gene is considered to be a particularly good tumor marker in that it tends to be quite specific for neoplastic cells and is identifiable in ‘discarded' next-generation sequencing reads that do not align to the reference sequence using standard bioinformatic tools.^84^ Low-level translocation detected by quantitative PCR could serve as a marker of minimal residual disease.

## EBV gene expression

Among 87 EBV-encoded mRNAs, 15 are highly expressed in the majority of EBV-positive gastric cancers according to the TCGA RNAseq data. Eight of these EBV transcripts (*BARF0*, *BALF3*, *BALF4*, *BALF5*, *A73*, *RPMS1*, *LF2* and *LF1*) are encoded in the *Bam*H1A region of the viral genome where the expressed viral miRs are also encoded.^10, 65, 85^ Although the medical literature suggests that latent membrane protein 1 is infrequently expressed in infected gastric adenocarcinoma by protein assays, TCGA RNAseq revealed that both EBV latent membrane protein encoding mRNAs (*LMP1*, *LMP2A*) were consistently expressed, albeit at low level. Latent membrane protein 2 or other viral gene products may contribute to hypermethylated DNA that is characteristic of infected cancers.^86^

Another interesting finding is consistent expression of EBV *BNLF2a*, which acts to inhibit the transporter associated with antigen processing, potentially thwarting antigen presentation.^87^ Also consistently expressed is the *BILF1* G-protein-coupled receptor that downregulates human leukocyte antigen class 1 protein expression.^88^ Both BNLF2a and BILF1 are thus involved in the cellular evasion of immune destruction, and are associated with the upregulation of the druggable natural killer/T-cell inhibitor IDO1 (indoleamine 2,3-dioxygenase 1) in EBV-positive tumors.^65^ As the IDO1 enzyme depletes tryptophan, it raises the question whether foods rich in tryptophan could overcome the effect. A viral gene of uncertain function, *BNLF2b*, is highly expressed, whereas *EBNA1* and *LF3* are expressed consistently but at low levels. The remaining latent and lytic viral genes (including the other EBNAs) are expressed at very low levels or only in a fraction of tumors.^10^

Selected viral microRNAs (encoded in the viral *Bam*H1A region of the viral genome) were highly expressed in all infected cancers in a fairly consistent pattern, according to the TCGA microRNA sequencing data.^10^ The most highly expressed of these EBV microRNAs are BART 10-5p, 7-3p and 7-5p. Emerging data reveals their impact on gastric carcinogenesis.^89, 90, 91^

## EBV genome structure and a rare chromosomal integration event

The EBV genome persists as an episome (double-stranded DNA of almost 172 kb) inside latently infected cells, with variable numbers of terminal repeat sequences within the circularized viral genome serving as a marker of clonality.^92^ The presence of clonal viral DNA in EBV-positive gastric cancers is evidence that infection precedes malignant transformation, a concept that is reinforced by histochemical evidence of *EBER* expression in all neoplastic cells of a given patient, and by evidence of EBV in some dysplastic lesions of the stomach mucosa.^93^ Whether EBV infection of epithelial cells occurs early or late during carcinogenesis is a subject of ongoing investigation.^94, 95, 96^

Multiple viral polymorphisms have been reported,^97, 98, 99, 100^ and the extent to which these mutations in the EBV genome impact gastric carcinogenesis is unknown. In the TCGA study, one gastric cancer had evidence of integration of the EBV genome into the human genome as revealed by multiple independent RNAseq reads revealing a fusion transcript predicted to join the first 20 amino acids of the human plasminogen receptor (*PLGRKT*, alias *C9orf46*) to almost the entire coding sequence of the early lytic EBV gene *BHLF1* (alias *EA-D*). In this particular tumor, *BHLF1* was expressed but the remaining viral gene expression pattern was not markedly different from other infected tumors. It should be noted that *PLGRKT* is located alongside *JAK2*, *CD274 (PD-L1)* and *PDCD1LG2 (PD-L2)* within the 9p24.1 locus that is commonly amplified in EBV-positive gastric cancers. Overall, the findings imply that viral integration can occur, is rare event or else does not does not frequently result in fusion transcripts, and may contribute to the activation of known oncogenes.

## Histopathology of EBV-positive gastric adenocarcinoma tissues

Prior work has shown that carcinoma with lymphoid stroma is characteristic of many EBV-positive cancers, although some infected cancers have more conventional tubular (intestinal) appearance by microscopy. Histologic classification of cancers using Lauren or World Health Organization schemes reveals that ‘genomically stable' cancers are enriched for diffuse histology^10^ (see Table 7). Further work should delve into histologic features differentiating the four molecular classes such as the character of lymphoid stroma and epithelial cell cytology/architecture (e.g. signet ring cell type, solid type *vs* isolated cell diffuse architecture as distinguished by Carneiro *et al.*^101^).

In EBV-positive cancers, the EBV genome is localized within the nucleus of malignant cells and is propagated to daughter cells during cell division. *EBER in situ* hybridization is considered the gold standard assay for assigning a tumor as ‘EBV-positive' by virtue of localization of abundantly expressed *EBER1* or *EBER2* long noncoding RNAs to malignant cells.^92^ High levels of EBV DNA (using quantitative PCR) or encoded viral RNA (using microarray, RNAseq or microRNA sequencing) are surrogate methods to distinguish EBV-positive cancers from EBV-negative cancers.^1, 10, 102^ High levels of EBV DNA are also seen during active viral infection (e.g. infectious mononucleosis, chronic active EBV infection, oral hairy leukoplakia), whereas low-level infection is commonly present in adult humans who were previously infected and then carry the virus for life in a small proportion of B lymphocytes.^92^

## Clinicopathologic features impacting treatment

From a clinical management perspective, it is clear that factors independent of molecular class impact treatment decisions at this time. Tumor stage is critical for devising plans for surgical resection, radiation and chemotherapy. *ERBB2 (HER2)* gene amplification qualifies metastatic cancer patients for HER2-directed antibody therapy.^103^ Yet, tumor stage, *ERBB2* status and survival were not strongly linked with molecular class,^10^ emphasizing that molecular class represents a conceptual biologic framework more than a practical tool at this time. Much remains to be learned about what appears to be four major routes of disease pathogenesis, and to capitalize on that understanding for purposes of diagnosis, management and prevention of cancer. Meanwhile, as most cancers have somatic variants in biochemical pathways that are putative targets for existing drugs, design of clinical trials is warranted to test the efficacy of targeted therapies.

## Implications for surgical pathologists and molecular pathologists

The reason why pathologists classify cancers is to promote clinical decision-making that improves patient outcomes. Classification criteria are periodically revised to incorporate new scientific evidence and methods of analysis. At this time, assignment to one of four molecular classes does not appear to add value beyond the features already actionable for clinical decision-making in gastric tumors (e.g. carcinoma *vs* gastrointestinal stromal tumor, *ERBB2 (HER2)* gene amplification in metastatic carcinoma, stage). Nevertheless, microscopic and ancillary tools can certainly be validated and applied to distinguish the four molecular subtypes at a reasonable cost, making it feasible to retrospectively and prospectively test clinical trial cohorts for molecular class.

The GastroGenus Gastric Cancer Classifier is a genomic assay that tests for EBV positivity, *MLH1* promoter methylation and sequences of selected cancer genes in the context of histopathologic classification. Results of the test in paraffin-embedded tumor tissue are interpreted with respect to molecular class and to find actionable gene variants in patients who fail standard therapy.^104^ Although therapy options for metastatic cancer patients remain limited and largely palliative,^103^ clinicians and patients increasingly demand genomic data by which to evaluate options for clinical trial enrollment, the results of which are likely to enhance understanding of pathogenesis and define the role of clinicopathologic tests in predicting drug efficacy.

Localization of EBV to malignant cells by *EBER in situ* hybridization remains the gold standard for assigning the EBV-positive class of cancers.^92^ EBV-positive gastric cancer patients qualify for enrollment in clinical trials of EBV-directed therapy (e.g. NCT00982449, NCT02080416). These trials use lytic induction therapy to convert infected cells from latent to replicative phases of viral infection, which is hypothesized to trigger cell death with potential for bystander killing of adjacent cells.^35, 48^

Also available are multiple trials of targeted therapy for gastric cancer having a particular gene defect or gene expression status. Among these trials are dozens for *ERRB2 (HER2)* gene defects (e.g. NCT01602406); NCT01613950 for *PIK3CA* mutation; NCT02016534 or NCT01874938 for *MET* gene amplification, NCT02052778 for *FGF* or *FGFR* gene defects; NCT02187783 for *CDK4*, *CDK6*, *CCND1*, *CCND3* or *CDKN2A* defects; NCT02022982 for *KRAS* mutation; and NCT01522820 for *CTAG1B* or *CTAG2* expression.

It is likely that the MSI molecular class of gastric cancers can be identified by virtue of MSI-high status (which requires testing of both tumor and germline DNA) or by MLH1 hypermethylation testing that requires only tumor tissue. Heretofore, MSI status has not generally been evaluated in gastric cancer patients except in the rare instance of suspected Lynch syndrome (e.g. personal or family history of pertinent cancer types, young age at diagnosis, blood relative with Lynch syndrome). The MSI test is used primarily to identify evidence of DNA mismatch repair defects in colorectal or endometrial cancer patients as part of Lynch syndrome screening.^105^ With the advent of massive parallel sequencing panels to identify germline variants in mismatch repair genes, it is likely that the MSI test will eventually be replaced by more cost-effective and direct means of testing for heritable cancer risk.^106, 107, 108^ For purposes of assigning molecular class of gastric cancer, *MLH1* methylation testing is likely to be a suitable surrogate test. A treatment trial of PD-1 antibody therapy (pembrolizumab, NCT01876511) currently enrolls patients with ‘MSI-positive' gastric cancer. Methylation-related silencing of MLH1 undoubtedly contributes to defective DNA repair and accumulating mutations in cancer tissue. *MLH1* promoter methylation also suggests better response to fluorouracil chemotherapy.

## Heritable predisposition to gastric cancer

Familial predisposition to gastric cancer is being examined in a clinical trial of subjects from high-risk families (NCT00172861). Pertinent hereditary cancer syndromes include Peutz-Jeghers (*STK11*), Li-Fraumeni (*TP53*), Lynch (*MLH1*, *MSH2*, *MSH6*, *PMS2*, *EPCAM*, *MUTYH)* and Cowden (*PTEN*) syndromes, as well as hereditary diffuse gastric cancer (*CDH1*).^109^ In the latter disorder, a hypomethylating drug is being considered to prevent cancer progression from premalignant lesions by thwarting *CDH1* promoter methylation. Table 8 lists genes reportedly associated with gastric cancer predisposition, some of which encode immune response factors that defend against EBV, *Hp* and other pathogens.

## Clinical implementation of genomic services

Well-validated genomic tests can provide robust, accurate and reproducible results that are powerful by virtue of the number of analytes that are evaluated, redundancy that boosts confidence in results, cross-species flexibility to detect pathogen-related human disease and a growing evidence base linking genetic findings to disease status in a manner that promotes favorable patient outcomes. Genomic tests typically analyze dozens to billions of targets in a single assay using microarrays or massive parallel sequencing. Performance studies of molecular technology have shown that genomic profiles of DNA, mRNA, methylation status and noncoding RNA can be rendered analytically sound and clinically informative for medical decision-making in clinical trials and ultimately in routine patient care.^110, 111, 112, 113, 114, 115, 116^

Building on decades of well-honed principles of laboratory medicine, pathologists and other laboratory professionals provide genomic services in clinical settings.^111^ Implementation is supported by (1) reliable commercial sources of reagents, supplies, instruments and software; (2) advances in biospecimen science promoting integrity of the nucleic acids input to the assays;^117^ (3) novel quality control reagents and methods to judge assay performance;^111, 118^ (4) informatics to facilitate interpretation of complex data generated from assays of patient and control specimens; (5) evidence of analytic and clinical performance; and (6) justification that the assay adds value (e.g. is faster, cheaper or more informative) than current means of diagnosis, monitoring, preventing or predicting efficacy of a given intervention in the pertinent population.

Each testing laboratory has a ‘standard operating procedure' that, along with a ‘validation report', establishes the evidence base substantiating indications for testing, specimen collection and processing, step-by-step analysis, quality control processes and guidance for interpreting data and for generating a report to the patient's medical record.^119^ Analytic interpretation is the process by which raw data is converted into reportable results. Clinical interpretation is the process by which a pathologist or other laboratory professional judges the medical significance of results in light of the clinical indication for which the service was ordered, the findings and the intended use of test results.

Analytic interpretation relies on thorough understanding of the technical strengths and weaknesses of the test system based on prior experience gathered during validation studies, literature review and subsequent clinical practice. Owing to test complexity and the need to interpret results in the context of the many quality assurance measures that are in place (including, in the case of molecular oncology specimens, histopathologic evaluation of the input lesion), it is clear that the resulting genomic sequence is not a stand-alone feature but rather a component of a package of technical and professional work constituting a medical service.

Interpretation is impacted by the level of confidence in results, as revealed by redundant findings (e.g. depth of coverage, mutant allele frequency, multiple tests of the same analyte or pathway, presence of both 5′ and 3′ ends of an mRNA) and the outcome of controls and quality checks (e.g. endogenous controls to evaluate adequacy of patient nucleic acid for the intended use, exogenous and spiked controls to evaluate run- and specimen-specific performance of the test system). Medical judgment is required to interpret findings in light of correlative clinical information (e.g. age, gender, tumor stage, histologic and immunohistochemical findings), published literature, validation work, genotype/phenotype databases and other reliable sources. Importantly, the interpretation must address the medical question posed by the ordering physician, and also consider incidental findings of importance to the patient and their blood relatives.

The report submitted to the patient's medical record documents findings and provides guidance to support clinical decision-making and follow-up. Such a report is best generated by a laboratory physician who is expert in disease pathobiology and in molecular technology, and who takes responsibility for the analyses and for the quality control processes supporting their interpretation.

In high complexity clinical laboratories in the United States (and in laboratories worldwide that are accredited by the College of American Pathologists or that meet equivalent standards), a laboratory physician must be available to discuss medical indications for testing and clinical implications of test results in a given patient. Published guidance outlines the principles of clinical grade genomic assay development, implementation and maintenance.^119, 120, 121, 122, 123^

## Summary

This article describes recent advances in understanding gastric carcinogenesis. The findings imply that gastric adenocarcinoma is four separate diseases with respect to molecular pathogenesis. Prior epidemiologic studies may have overlooked the EBV molecular class that comprises only 9% of cancers, while emphasizing the chromosome instability class that comprises 50% of cancers. Furthermore, historic studies lumped cases with CpG island hypermethylation into a single group, but emerging evidence reveals the distinct patterns of hypermethylation in EBV-positive *vs* MSI molecular classes of cancer. Cancers of diffuse histology tend to be ‘genomically stable' implying few mutations or gene amplifications, and prevalent defects in RHO signaling downstream of G-protein-coupled receptors.

Pathologists increasingly have the tools to support clinical trials of targeted therapy and to advance routine health care using ancillary molecular tests. The ‘GastroGenus Gastric Cancer Classifier' is an example of a genomic test panel that adds value beyond histopathology of formalin-fixed cancer tissue by evaluating EBV status, *MLH1* promoter methylation and multiple cancer gene sequences to interpret molecular class and to identify actionable mutations revealing clinical trial options or off-label use of an existing drug for metastatic cancer patients who fail standard therapy. On the horizon is integrative genomic technology combining gene expression profiles with DNA/RNA sequence information such as mutation, splice variants, fusion and pathogen identification. Advanced genomic technology shows promise to assist in early diagnosis, classification and monitoring of affected patients. Hopefully cancer prevention is also among the ways that genomic technology might improve public health.

## Figures and Tables

**Table 1:** Key genomic characteristics in the four molecular classes of gastric adenocarcinoma proposed by The Cancer Genome Analysis Network

*Epstein–Barr virus positive (9% of gastric cancers)*
* PIK3CA* mutation
* *Marked DNA hypermethylation including in *CDKN2A (p16)* but not *MLH1* promoters
* JAK2* gene amplification
* *Immune response gene dysregulation with *CD274* and *PDCD1LG2* (*PD-L1 and PD-L2)* amplification and overexpression

*Microsatellite instability (22% of gastric cancers)*
* *Extensive DNA hypermethylation with epigenetic silencing of *MLH1*
* *Hypermutation of many genes including HLA class 1 factors affecting antigen presentation

*Genomically stable (20% of gastric cancers)*
* *Unique GTPase-activating mutations or fusions (RHOA or ARHGAPs)
* CDH1* (E-cadherin) mutation (somatic)
* *Diffuse histologic subtype

*Chromosome instability (50% of gastric cancers)*
* *Multiple gene amplifications and deletions—notably *EGFR, VEGFA* and other receptor tyrosine kinase gene amplification, or cell cycle regulatory gene amplification (*CCND1, CCNE1, CDK6*)
* TP53* mutation

Abbreviation: HLA, human leukocyte antigen.

**Table 2:** Top 10 most dysregulated human mRNAs and microRNAs in gastric cancer tissue compared with non-malignant mucosa^a^

*EBV+*		*MSI*		*Genomically stable*		*Chromosome instability*	
*Upregulated*	*Downregulated*	*Upregulated*	*Downregulated*	*Upregulated*	*Downregulated*	*Upregulated*	*Downregulated*
CST1	GKN1	CST1	GKN1	SFRP4	GKN1	CST1	GKN1
CXCL9	GKN2	CLDN3	GKN2	CLDN3	GKN2	CLDN3	GKN2
CXCL10	REG3A	CDH17	PGC	THBS4	LIPF	VIL1	LIPF
CXCL11	TFF1	SPP1	LIPF	THBS2	PGC	SFRP4	GIF
UBD	LIPF	COL10A1	REG3A	CST1	TFF2	CLDN1	PGA3
IDO1	TFF2	IL8	GIF	BGN	GIF	CDH17	REG3A
MMP7	PSCA	CXCL1	PGA3	FNDC1	REG3A	MUC3A	CHGA
CLDN1	PGC	SULF1	CHGA	COL8A1	PGA3	MMP11	IGJ
APOC1	GIF	CXCL9	CXCL17	ASPN	PSCA	COL10A1	KRT20
OLFM4	PGA3	MMP11	KRT20	SULF1	CXCL17	INHBA	PGC
							
*MicroRNAs:*							
Mir-142	Mir-375	Mir-196b	(none were significant)	Mir-196a	Mir-451	Mir-196a	Mir-1
Mir-335	Mir-1	Mir-196a	Mir-196b	Mir-1	Mir-196b	Mir-133a	
Mir-146b	Mir-133a	Mir-210	Mir-217	Mir-486	Mir-135b	Mir-145	
Mir-21	Mir-9	Mir-194-2	Mir-21	Mir-144	Mir-194-2	Mir-145	
Mir-501	Mir-145	Mir-429	Mir-708	Mir-9	Mir-192	Mir-139	
Mir-146b	Mir-139	Mir-200a		Mir-146b	Mir-133a	Mir-194	Mir-451
Mir-455	Mir-451	Mir-183		Mir-146b	Mir-29c	Mir-21	Mir-9
Mir-181a-1	Mir-145	Mir-194		Mir-181a-1	Mir-145	Mir-501	Mir-486
Mir-181b	Mir-486	Mir-192		Mir-542	Mir-365	Mir-335	Mir-29c
Mir-19a	Mir-29c	Mir-182		Mir-199b	Mir-139	Mir-183	Mir-143

Abbreviations: EBV, Epstein–Barr virus; MSI, microsatellite instability.

a Ranked in order of fold change in mRNA or microRNA level, with highest fold change at the top.

**Table 3:** Methylated gene silencing in EBV-positive compared with EBV-negative gastric cancers^a^

*Gene silenced in EBV+ cancers*	*Also silenced in EBV(−)gastric cancers (%)*	*Gene silenced in EBV+ cancers*	*Also silenced in EBV(−)gastric cancers (%)*
*RCOR2*	0	*NHLRC1*	25
*RHOF*	1	*TSPY26P*	28
*TMEM52*	1	*KIAA1383*	29
*CLDN3*	1	*ZNF530*	32
*HOXA10*	1	*KRT7*	32
*CRAT*	3	*ARHGEF10*	32
*FNDC4*	4	*PRDM5*	42
*PRKCDBP*	4	*THNSL2*	42
*BMP8B*	6	*RAB34*	44
*TXNRD3*	7	*CHST10*	49
*LDLRAD3*	8	*TP73-AS1*	49
*B3GALNT1*	9	*ZNF813*	50
*ESYT3*	10	*ZNF549*	53
*OSBP2*	10	*ZNF470*	61
*C2CD4B*	12	*ZNF518B*	61
*MAP1LC3A*	12	*HOXA1*	63
*C5orf42*	14	*LOC339803*	64
*SOGA1*	14	*PCDHGC5*	64
*SCRN1*	16	*TSPYL5*	68
*C8orf47*	20	*ZNF610*	68
*TPD52L1*	22	*ZFP28*	70
		*ZNF542*	76

Abbreviations: EBV, Epstein–Barr virus; TCGA, The Cancer Genome Atlas.

a Each gene was silenced in at least 95% of EBV-positive gastric cancers, as reported by the TCGA Network.

**Table 4:** Strategies for virus-directed therapy

*Enhance immune response to infected cells*
-Infuse virus-directed cytotoxic T cells
-Upregulate viral antigen presentation
-Activate T cells (e.g. *PD-L1* inhibitor)
-Reduce immune tolerance

*Enhance immunogenicity of infected cells*
-Induce lytic viral gene expression

*Reverse virus-mediated cell maintenance*
-Block viral drivers of growth, survival and immune evasion
-Alter viral epigenetic controls (methylation, acetylation)

Trigger apoptosis of infected cells
-The natural end point of lytic viral infection
-Viral protein activates a toxin (gancyclovir)

**Table 5:** Genes commonly mutated in gastric cancer

*All molecular classes*^a^			
*TP53*	*KRAS*	*RNF43*	*RASA1*
*CDH1*	*MUC6*	*ABCA10*	*FAM46D*
*SMAD4*	*APC*	*CTNNB1*	*PLB1*
*PIK3CA*	*BCOR*	*MACF1*	*CNGA4*
*RHOA*	*EYA4*	*SMAD2*	*EIF2C4*
*ARID1A*	*BNC2*	*SOHLH2*	*ERBB2 (HER2)*
			*PTPRC*
			
*EBV-positive class vs EBV-negative classes*			
*PIK3CA*	*GRIK1*	*BCOR*	*ARID1A*
*TCHH*	*WNK1*	*MAMLD1*	

Abbreviation: EBV, Epstein–Barr virus.

a Hypermutated cancers were excluded when compiling this list.

**Table 6:** Gene regions commonly amplified or deleted in EBV-positive gastric cancers^a^

	*Amplified loci*		
	*9p24.1*	*17q12*	*11p13*
	*INSL4*	***ERBB2 (HER2)***	***CD44***
	***JAK2***	*PNMT*	*SLC1A2*
	*RLN1*	*TCAP*	*FJX1*
	*RLN2*	*PGAP3*	*PAMR1*
	*INSL6*		
	***CD274 (PD-L1)***		
	*PLGRKT (C9orf46)*		
	***PDCD1LG2 (PD-L2)***		

	*Deleted loci*					
	*16q23.1*	*7q31.1*	*9p24.1*	*4q22.1*	*10q23.31*	*20p12.1*
	***WWOX***	*LRRN3*	***PTPRD***	***FAM190A***	***PTEN***	*FLRT3*
		***IMMP2L***			*ATAD1*	***MACROD2***
					*CFL1P1*	*MACROD2-AS1*
					*KLLN*	

Abbreviation: EBV, Epstein–Barr virus.

a Proposed oncogenes and tumor suppressor genes are shown in bold.

**Table 7:** Histopathologic characteristics of the four molecular classes of gastric adenocarcinoma^a^

	**n**	*All cancers (%)*	*Epstein–Barr virus positive (%)*	*Microsatellite instability (%)*	*Chromosome instability (%)*	*Genomically stabile (%)*
*Sex*						
Female	113	38	4	32	44	20
Male	182	62	12	15	53	20
						
*Lauren classification*						
Diffuse	69	23	7	9	26	58
Intestinal	196	66	8	25	60	8
Mixed	19	6	16	16	53	16
Not specified	11	4	27	64	9	0
						
*WHO classification*						
Mixed	19	6	16	16	53	16
Mucinous	18	6	0	39	50	11
Papillary	22	8	5	18	68	9
Poor cohesive	69	23	7	9	26	58
Tubular	140	48	6	25	62	6
Not specified	27	9	30	33	30	7
						
*Anatomic site*						
Antrum	114	39	5	27	43	25
Body	116	39	14	22	49	16
Proximal	57	19	7	9	65	19
Not specified	8	3	0	38	50	13

Abbreviations: TCGA, The Cancer Genome Atlas; WHO, World Health Organization.

a Data from the TCGA Network.

**Table 8:** Genes reportedly associated with heritable predisposition to gastric cancer

*APC*	*CDH1*	*FASLG*	*IL6*	*MUC2*	*PRKAA1*	*TLR1*
*ATM*	*CHEK2*	*GSTM1*	*IL8*	*MUTYH*	*PSCA*	*TLR2*
*GJA4*	*CTNNA1*	*GSTP1*	*LEPR*	*NOS2*	*PTEN*	*TLR4*
*MSH6*	*CTNNB1*	*GSTT1*	*LTA*	*NQO1*	*PTGS2*	*TLR9*
*APC*	*CYP2E1*	*IFNGR1*	*MET*	*PARP1*	*PTPN11*	*TNF*
*APOE*	*DNMT3A*	*IL10*	*MIF*	*PLCE1*	*SMAD4*	*TP53*
*BIRC5*	*EPCAM*	*IL17A*	*MLH1*	*PMS2*	*SOD2*	*TYMS*
*BMPR1A*	*ERCC1*	*IL1B*	*MSH2,*	*POLD1*	*SPP1*	*VEGFA*
*BRCA1*	*ERCC5*	*IL1RN*	*MTHFR*	*POLE*	*STK11*	*WWOX*
*BRCA2*	*FAS*	*IL4R*	*MUC1*	*PPARG*	*TGFB*	*XRCC1*

## Notes

MLG serves on an advisory board of Illumina and received assay validation reagents from Illumina.
